# The Cerato-Platanin protein Epl-1 from *Trichoderma harzianum* is involved in mycoparasitism, plant resistance induction and self cell wall protection

**DOI:** 10.1038/srep17998

**Published:** 2015-12-09

**Authors:** Eriston Vieira Gomes, Mariana do Nascimento Costa, Renato Graciano de Paula, Rafael Ricci de Azevedo, Francilene Lopes da Silva, Eliane F. Noronha, Cirano José Ulhoa, Valdirene Neves Monteiro, Rosa Elena Cardoza, Santiago Gutiérrez, Roberto Nascimento Silva

**Affiliations:** 1 Department of Biochemistry and Immunology, Ribeirão Preto Medical School, University of São Paulo, Ribeirão Preto, SP, Brazil; 2 Department of Molecular and Cellular Biology and Pathogenic Bioagents, Ribeirão Preto Medical School, University of São Paulo, Ribeirão Preto, SP, Brazil; 3 Department of Cellular Biology, University of Brasilia, Brasília, Distrito Federal, Brazil; 4 Department of Biochemistry and Cellular Biology, Biological Sciences Institute, Federal University of Goias, Goiânia, Goiás, Brazil; 5 Department of Biochemistry, State University of Goias, Anápolis, Goiás, Brazil; 6 Department of Microbiology, University School of Agricultural Engineers, University of León, Ponferrada, Spain

## Abstract

*Trichoderma harzianum* species are well known as biocontrol agents against important fungal phytopathogens. Mycoparasitism is one of the strategies used by this fungus in the biocontrol process. In this work, we analyzed the effect of Epl-1 protein, previously described as plant resistance elicitor, in expression modulation of *T*. *harzianum* genes involved in mycoparasitism process against phytopathogenic fungi; self cell wall protection and recognition; host hyphae coiling and triggering expression of defense-related genes in beans plants. The results indicated that the absence of Epl-1 protein affects the expression of all mycoparasitism genes analyzed in direct confrontation assays against phytopathogen *Sclerotinia sclerotiorum* as well as *T*. *harzianum* itself; the host mycoparasitic coiling process and expression modulation of plant defense genes showing different pattern compared with wild type strain. These data indicated the involvement *T*. *harzianum* Epl-1 in self and host interaction and also recognition of *T*. *harzianum* as a symbiotic fungus by the bean plants.

Chemical pesticides have long been used to protect crops from diseases. Fungicides and fumigants are often applied in greater quantities than herbicides and insecticides in agricultural practices and have adverse effects on the environment and human health. Thus, reduction or elimination of synthetic pesticide in agriculture is highly desirable. Hence, biocontrol agents are being used to control phytopathogenic fungi and to induce natural resistance in plants against these organisms. Further, these agents can be integrated with reduced chemical doses for controlling plant pathogens, resulting in minimal environmental impact^1^,^2^.

Proteins of the Cerato-platanin (CP) family are released during the early developmental stages of filamentous fungi. They can act as elicitors and induce defense responses in plants. For example Sm1 or Epl-1 proteins from *Trichoderma virens* and *T. atroviride*, respectively^3^,^4^,^5^,^6^ have been shown to induce phytoalexin production and/or cell death in host and non-host plants.^7^,^8^,^9^

*Trichoderma* species are widely spread used as biocontrol agents in agriculture and forestry; they can induce a combination of antagonist mechanisms such as antibiosis via the production of secondary metabolites having antifungal activity^10^,^11^,^12^,^13^; mycoparasitism, which involves the production of cell wall-degrading enzymes, competition for nutrients or space; and induction of resistance in plants via the production and secretion of elicitor molecules^14^,^15^,^16^. Among the *Trichoderma* species, *T*. *harzianum* is considered as one of the most effective biocontrol agents^17^.

In this study, the role of *T*. *harzianum* Epl-1 protein during the interaction with pathogens and plants was analyzed, especially its involvement in the mycoparasitism process. The effect of the absence of this elicitor on the induction of pathogen resistance in bean plants, as well as its role in cell wall protection and host and self-recognition was investigated.

## Results

### The *in silico* analyses of *T*. *harzianum* Epl-1: Features and probable regulation network

Our *in silico* analyses, revealed that *T*. *harzianum epl-1* gene (ID: 508110; open reading frame (ORF), 417 bp; intron, 64 bp) encodes a protein of 138 amino acids. According to Expasy program ProtParam^18^, the protein has a theoretical molecular weight of 14,356.1 Da; and isoeletric point of 6.23 with an instability index of 21.74, suggesting that it is stable. The first 18 amino acid residues correspond to a secretion signal peptide. Analyses of Epl-1using NetOGlyc 3.1 software, indicated a potential *O*-glyosylation (Supplementary Figure 1), but no potential *N*-glycosylation sites (data not shown). In addition, seven potential *O*-(beta)-*N*-acetyl-glycosylation sites (YinOYang 1.2 software)^19^ (Supplementary Figure 2) and five phosphorylation sites (Supplementary Figure 3) were observed.

The presence of putative regulatory motifs in *T*. *harzianum epl-1* was investigated by analyzing 2.105 bp region upstream of the ATG initiation codon and 2.000 bp downstream of the ATT termination codon by using SCOPE software^20^ (Supplementary Figure 4). Analysis of the 5′ region reveled two TATAA Box sites and fourteen CAAT box sites related to transcription initiation^21^. Only one of the four sequences required for the putative binding of regulatory proteins involved in mycoparasitism (MYC)^22^ (MYC1 5′-GCTTCA-3′) was found in the *T*. *harzianum epl-1* promoter region. This region also contained six copies of consensus sequence (5′-SYGGRG - 3′) which is associated with the binding of *Aspergillus nidulans* carbon catabolic repressor CREA^23^,^24^. One copy of the motif 5′-HGATAR-3′ which mediates global nitrogen regulation^25^,^26^,^27^; three copies of motif 5′-GCCARG-3′, which is the recognition site for the pH regulatory protein PacC in *Aspergillus* spp^21^ and the pentanucleotide CCCCT (C4T) which is related to stress response element regulation in fungi^22^,^28^,^29^ were found in the *epl-1* promoter. The analysis of the 3′ region allowed the identification of five putative polyadenylation sites without any TAAATAA motif.

### *T*. *harzianum epl-1* gene deletion, recovery and transformant selection

Knockout mutants of *epl-1* were produced using double-crossover homologous recombination. Protoplasts of *T*. *harzianum* (ALL42) were transformed with a linear DNA sequence containing a hygromycin B expression cassette flanked by two DNA fragments homologous to the *epl-1* 5′ and 3′ regions. Polymerase chain reaction (PCR) analysis of the epl-1 gene region from mitotically stable transformants showed bands corresponding to the wild-type gene (1.090 bp), complete deletion cassette (3.457 bp) and the gene for hygromycin B resistance (*hph* 600 bp) (Supplementary Figure 5A, 5B, and 5C, respectively). Six transformants that showed bands only for the complete deletion cassette and *hph* were selected and one of them was subjected for Southern blot analysis (Supplementary Figure 6). The total DNA isolated and digested with *EcoRV* endonuclease showed two hybridization patterns for the wild-type strain (800 bp and approximately 1.500 bp; Supplementary Figure 6A,C) and one band pattern for the mutant strain (approximately 4.500 bp; Supplementary Figure 6B,C). The *epl-1* deletion (∆epl-1) was also confirmed by real-time quantitative PCR (RT-qPCR) analysis. Subsequently, the mutant *T*. *harzianum* ∆*epl-1* strain was complemented with the epl-1 ORF fused with the green fluorescent protein (*GFP*) gene. The *T*. *harzianum epl-1* complemented (RecEpl-1-GFP) transformants were selected by their ability to grow in minimal medium, uridine and uracil free; the GFP signal in fluorescence microscopy images; PCR and electrophoreses of the amplification product of *epl-1* encoding gene; and RT-qPCR analysis of the *epl-1* relative expression from all *Trichoderma* strains, as described in Materials and Methods section (Supplementary Figure 7A–D).

### Epl-1 protein is not essential for *T*. *harzianum* antagonistic potential

**Figure 1:**
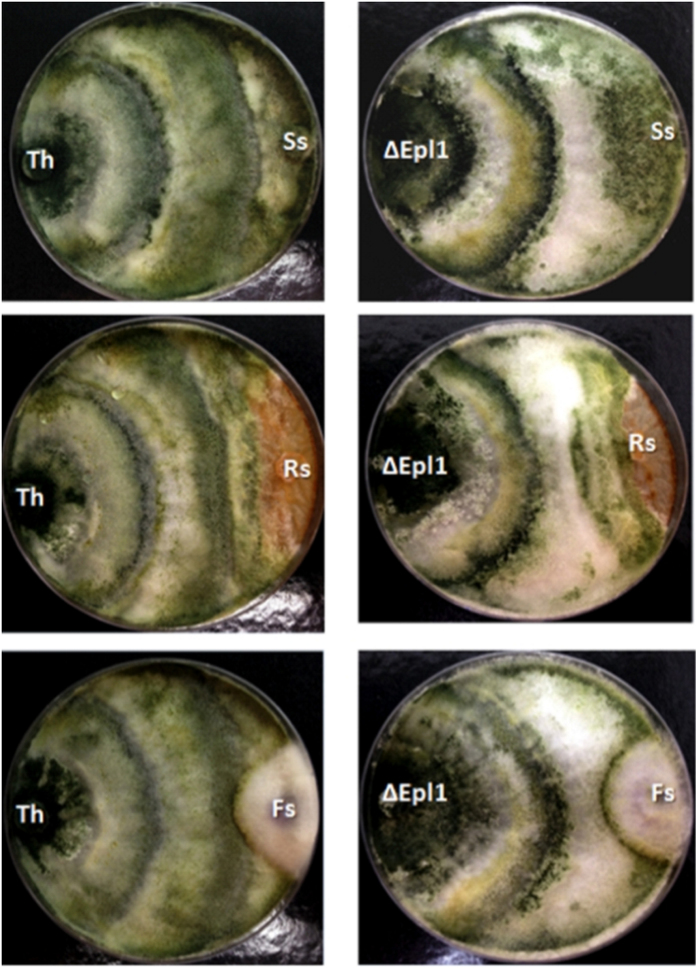
*In vitro* antagonistic potential assay. **(Th)** *T*. *harzianum* wild type; **(∆Epl1)** *T*. *harzianum* ∆*epl-1*; **(Ss)** *S. sclerotiorum*; **(Rs)** *R. solani*; **(Fs)** *F. solani*.

*In vitro* antagonistic activity of the *Trichoderma* strains against three plant pathogens (*Sclerotinia sclerotiorum*, *Rhizoctonia solani* and *Fusarium solani*) was analyzed as described by Bell *et al.*^30^ (modified method), and *Trichoderma* strains were classified as “efficient” to antagonize the plant pathogens if the mean score was <3, “moderate” if the mean score was 3 and “inefficient” if the mean score was >3 (Supplementary Figure 8). The deletion of *epl-1* did not affect the ability of *T*. *harzianum* to antagonize plant pathogens, indicating that Epl-1 was not essential for this function. Wild-type and deletion mutants were efficient against all the pathogen strains tested, showing hyperparasitism signals; further a discrete antagonism improvement was noted in the mutant against *R. solani* compared to that of the wild-type strain (Fig. 1).

### *T*. *harzianum* Epl-1 protein is carried by vesicles and predominantly transported to the fungal cell wall

**Figure 2:**
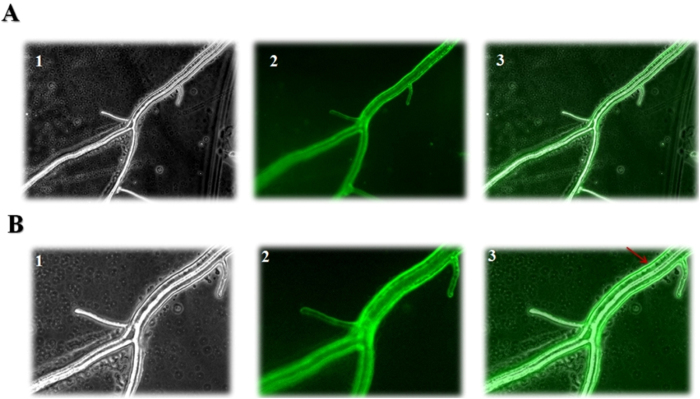
Fluorescence microscopy analyses of Epl-1 fused with GFP. (**A**) – Fluorescence microscopy of *Trichoderma harzianum* RecEpl-1-GFP hyphae in 20× optical magnification. 1 – Light field; 2 – GFP; 3 – Merge. (**B**) – Fluorescence microscopy of *Trichoderma harzianum* RecEpl-1-GFP strain hyphae in 40× optical magnification. 1 – Light field; 2 – GFP; 3 – Merge. The arrow indicates the predominance of Epl-1 in the cell wall region and its transport through the hyphae by vesicles.

Fluorescence microscopy analyses of *T*. *harzianum* strain expressing Epl-1 fused with GFP showed a dynamic flow of vesicles containing Epl-1 through the *Trichoderma* hyphae in the presence of *S. sclerotiorum*. The vesicles fused with the plasma membrane and accumulated predominantly in the *Trichoderma* cell wall (Fig. 2 and Supplementary Videos 1 and 2).

### *T*. *harzianum* Epl-1 protein is involved in hyphal recognition and mycoparasitic hyphal coiling process

**Figure 3:**
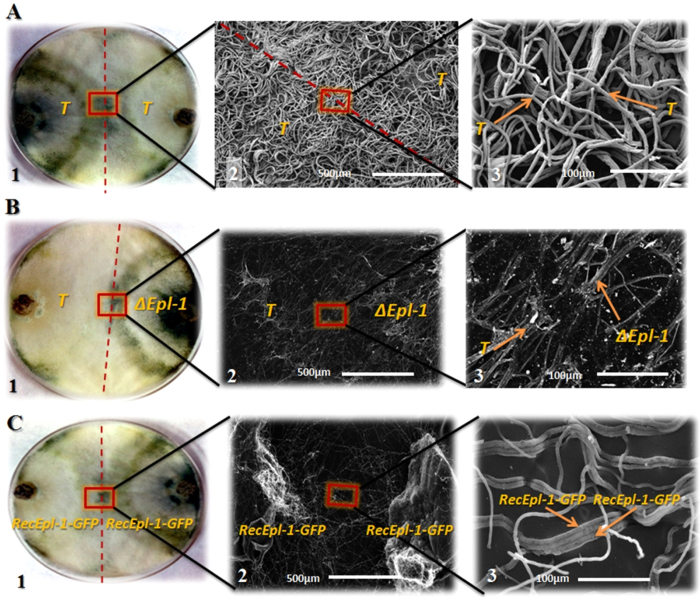
Scanning electron microscopy analysis (SEM). (**A**) - SEM analysis of the interaction between *T*. *harzianum* wild type (*T*) strains. **1** – *T*. *harzianum* wild type dual culture plate. **2** – SEM analysis of *T*. *harzianum* wild type strains interaction. 2.5 kV acceleration voltage, magnification 250×. **3** – SEM analysis of *T*. *harzianum* wild type strains. 2.5 kV acceleration voltage, magnification 500×. (**B**) - SEM analysis of the interaction between *T*. *harzianum* wild type (*T*) and *T*. *harzianum* ∆*epl-1* (∆*epl-1*) strains. **1** – *T*. *harzianum* wild type and mutant ∆*epl-1* strains dual culture plate. **2** – SEM analysis of *T*. *harzianum* wild type and mutant ∆*epl-1* interaction. 30 kV acceleration voltage, magnification 350×. **3** – SEM analysis of *T*. *harzianum* wild type and mutant ∆*epl-1* interaction. 30 kV acceleration voltage, magnification 500×. (**C**) - SEM analysis of the interaction between *T*. *harzianum* RecEpl-1-GFP strains (RecEpl-1-GFP). **1** – *T*. *harzianum* RecEpl-1-GFP strains dual culture plate. **2** – SEM analysis of *T*. *harzianum* RecEpl-1-GFP strains interaction. 30 kV acceleration voltage, magnification 350×. **3** – SEM analysis of *T*. *harzianum* RecEpl-1-GFP strains interaction. 30 kV acceleration voltage, magnification 500×.

**Figure 4:**
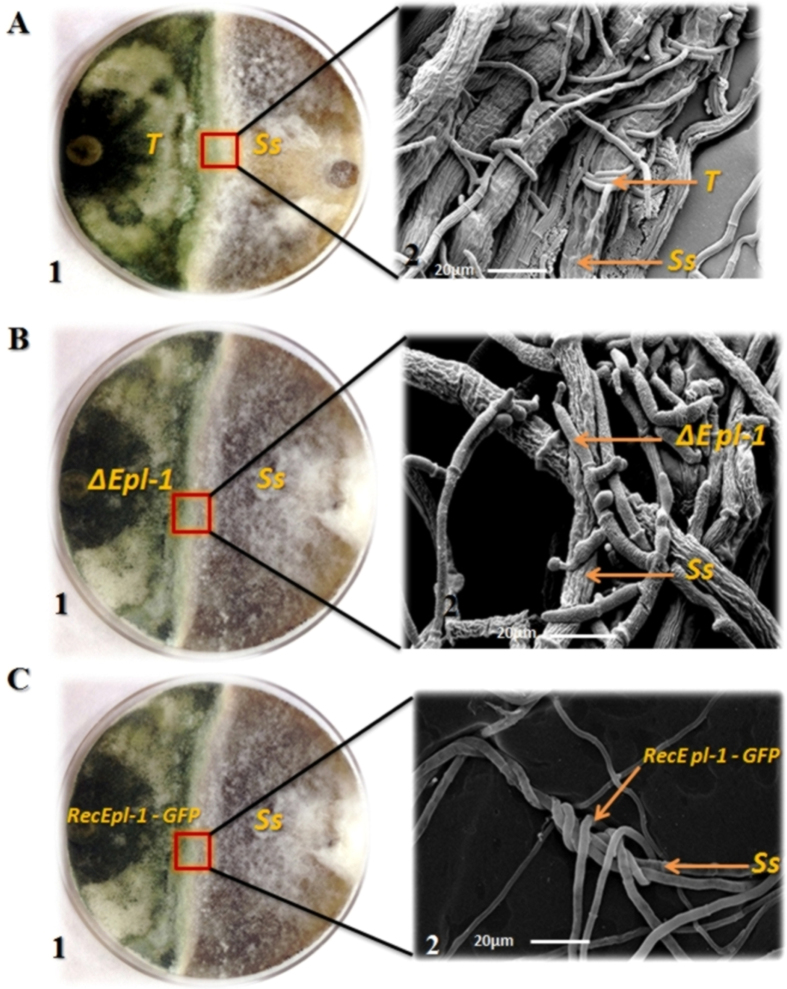
Scanning electron microscopy analysis (SEM). (**A**) - SEM analysis of the interaction between *T*. *harzianum* wild type (*T*) and *S. sclerotiorum* (*Ss*) strains. **1** – *T*. *harzianum* wild type and *S. sclerotiorum* dual culture plate. **2** – SEM analysis of *T*. *harzianum* wild type and *S. sclerotiorum* strains interaction. 5 kV acceleration voltage, magnification 900×. (**B**) - SEM analysis of the interaction between *T*. *harzianum* ∆*epl-1* (∆*epl-1*) and *S. sclerotiorum* (*Ss*) strains. **1** – *T*. *harzianum* ∆*epl-1* and *S. sclerotiorum* dual culture plate. **2** – SEM analysis of *T*. *harzianum* ∆*epl-1* and *S. sclerotiorum* strains interaction. 5 kV acceleration voltage, magnification 900×. **C** - SEM analysis of the interaction between *T*. *harzianum* RecEpl-1-GFP (RecEpl-1-GFP) and *S. sclerotiorum* (*Ss*) strains. **1** – *T*. *harzianum* RecEpl-1-GFP and *S. sclerotiorum* dual culture plate. **2** – SEM analysis of *T*. *harzianum* RecEpl-1-GFP and *S. sclerotiorum* strains interaction. 5 kV acceleration voltage, magnification 900×.

Interaction between *T*. *harzianum* strains and *S. sclerotiorum* was analyzed using scanning electron microscopy (SEM). In the *T*. *harzianum* wild-type interaction zone, a homogeneous mycelial mass without a clear division between both the isolates was observed with no hyphal degradation (Fig. 3A). In the interaction zone between wild-type *T*. *harzianum* and mutant ∆*epl-1* strains, a clear interaction line separating both the strains and debris from probable enzymatic hyphal degradation were noted, indicating non-hyphal recognition between both the isolates (Fig. 3B). The self recognition feature was reestablished in RecEpl-1-GFP complemented strain, which showed parallel hyphal growth between the two strains and no degradation debris was noted in the wild-type interaction (Fig. 3C). In the interaction between wild-type *T*. *harzianum* and *S. sclerotiorum*, the typical *Trichoderma* mycoparasitism process with hyphal coiling around the host was observed (Fig. 4A). Interestingly, the mycoparasitical interaction process could be observed between *T*. *harzianum* ∆*epl-1* and *S. sclerotiorum*; the typical *Trichoderma* hyphal coiling was not noted (Fig. 4B). Further, the coiling capacity was reestablished in the *T*. *harzianum* RecEpl-1-GFP-complemented strain (Fig. 4C), indicating that the Epl-1 interferes with the *Trichoderma* mycoparasitic process. Similar results were observed during the interaction between *T*. *harzianum* strains and *R. solani* (data not shown).

### Absence of Epl-1 affects expression levels of mycoparasitism genes in direct confrontation

**Figure 5:**
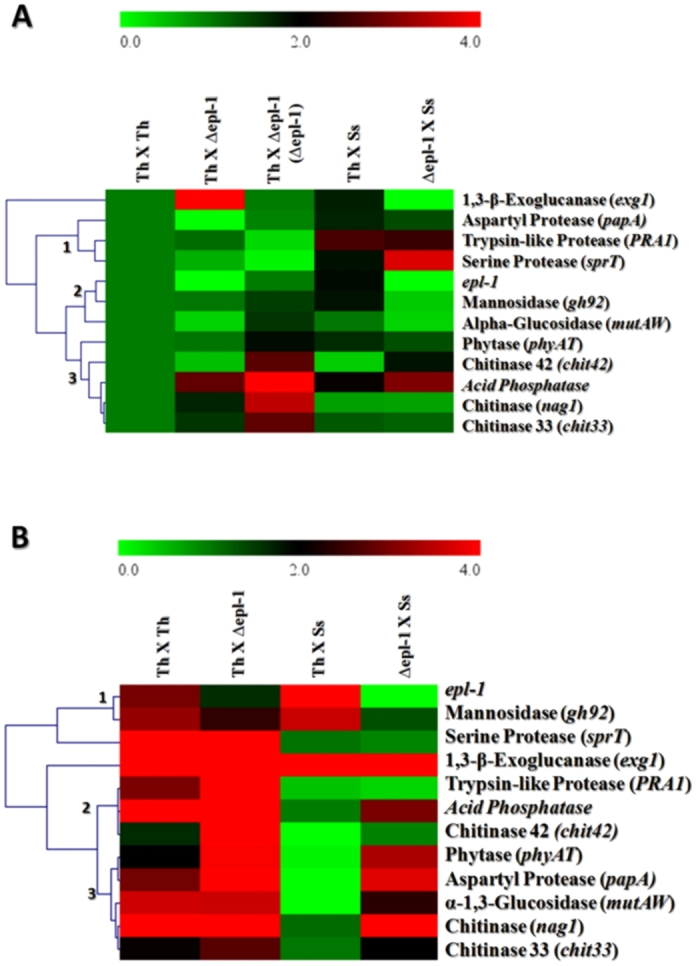
Heat map and cluster categorization of *T. harzianum* mycoparasitism-related genes expression. (**A**) – Heat map of qPCR expression analyzes of mycoparasitism genes before hyphae contact. (**B**) - Heat map of qPCR expression analyzes of mycoparasitism genes after hyphae contact. Th – *T. harzianum* wild type; ∆epl-1 – *T*. *harzianum* ∆*epl-1*; Ss – *S. sclerotiorum*.

Direct confrontation assays were performed between *T*. *harzianum* and *S. sclerotiorum* strains. Mycelial RNAs from the confrontation area were extracted before (BC), during (C), and after (AC) hyphae contact (Supplementary Figure 9). The relative expression levels of *epl-1* and eleven mycoparasitism gene markers were analyzed to determine the effect of Epl-1 on the modulation of mycoparasitism gene expression. In the confrontation between wild-type *T*. *harzianum* and mutant ∆*epl-1* strains, eight genes were up-regulated, of which *exg1*, *acid phosphatase*, *chit42*, *papA*, and *PRA1* were more prominent (Supplementary Figures 10 and 11). For the confrontation between wild-type and mutant ∆*epl-1 Trichoderma* strains and *S. sclerotiorum*, all analyzed genes showed remarkable difference in their expression levels, indicating an altered perception of the host and itself (Supplementary Figures 10 and 11). Gene expression was not significantly different (p > 0.05) between C and AC conditions (data not shown). The qPCR expression data were used to generate heat maps for determine the effects of *epl-1* deletion on mycoparasitism gene expression modulation by using Mev MultiExperiment Viewer Software^31^. In the BC condition, despite the relatively stable gene expressions with slight variations, significant differences were noted in the expression modulation of genes in all the analyzed conditions (Fig. 5A). However, during AC, the difference in gene expression modulation became more evident, as observed between wild-type and mutant *Trichoderma* strains and *Trichoderma vs. Sclerotinia* confrontations (Fig. 5B). Another relevant finding was the gene clustering according to their expression pattern. For the BC and AC conditions three distinct groups were formed: Group 1 (*PRA1* and *sprT*), Group 2 (*epl-1* and *gh92*) and Group 3 (*nag1* and *chit33*) for BC (the remaining genes showed different expression profiles; Fig. 5A). Group 1 (*epl-1* and *gh92*); Group 2 (*PRA1*, *acid phosphatase* and *chit42*) and Group 3 (*phyAT*, *papA*, *mutAW*, *nag1*, and *chit33*) for AC (Fig. 5B).

### Expression analysis of defense-related genes in bean plants was modified by Epl-1

**Figure 6:**
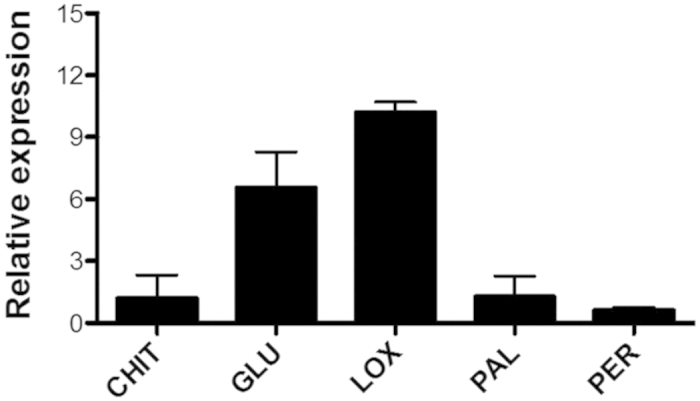
*T. harzianum* ∆*Epl-1*Mutant/Wild Type Fold change analysis of defense-related genes expression (linear) in common bean plant roots 24 hours after *T. harzianum* strains inoculation. *bch1* – chitinase; *glu1* - β-1,3-glucanase; *lox1* – lypoxygenase*; pal* – phenylalanine ammonia lyase; *pod3 –* peroxidase. The data were presented using the 2^−ΔΔCt^ method.

In this study, the effects of *T*. *harzianum* Epl-1 in eliciting the expression of five defense-related genes in bean plants was analyzed 24 h after inoculation of both, wild-type and ∆*epl-1* mutant *T*. *harzianum* strains. Plants without *Trichoderma* inoculation were used as controls. Plants inoculated with the **Δ*epl-1* mutant and wild-type strains showed the up-regulation of *lox* and *glu* genes (19.1- and 6.5-fold respectively; Fig. 6). *Trichoderma* inoculation significantly induced *pod3* gene at 72 h (data not shown).

## Discussion

The biochemistry and genetics of the mycoparasitism process has been widely studied in several species of *Trichoderma*, because of their importance as pest biocontrol agents in agriculture. Various enzymes, proteins, and effectors involved in fungi recognition and mycoparasitc responses have been identified^32^,^33^,^34^. In this study, *T*. *harzianum* Epl-1 protein, a virulence factor that induces local and systemic defenses in plants, was found to be important during the interaction with itself and with different host organisms and played an essential role in the mycoparasitism process, by affecting *T*. *harzianum* self-recognition, regulating mycoparasitism-related gene expression, modulating mycoparasitic hyphal coiling and regulating plant defense-related gene induction.

Proteins of the CP family are involved in fungal growth, development, recognition, adhesion, cell-wall morphogenesis^35^, antagonism^36^ and parasitism^37^. However, data on the primary function of CPs are lacking. The CP member analyzed in this study showed 100% identity with Epl-1 from *Trichoderma viride* (*Hypocrea rufa*) and 94% identity with Sm1 from *T*. *harzianum* described by Freitas *et al.*^38^. Frischmann *et al.*^36^ suggested the presence of three *epl* genes in *Trichoderma atroviride* genome: *epl-1* was predominantly expressed during hyphal growth, *epl-2* was mainly expressed during the spore formation and *epl3* was not highly expressed during the growth. They suggested that single and double gene knockouts of *epl-1* and *epl-2* did not remarkably affect the growth and development of the fungus. Their high homology across different species and the presence of some of them in fungal cell wall suggest that they might be associated with the lifestyle of fungi^36^. The *epl-1* gene expression was strongly induced, especially during C condition, consistent with the findings for Epl-1 expression during the confrontation between *T. atroviride* and *R. solani*^5^. Thus, we constructed the *T*. *harzianum* ∆*epl-1* mutant strain to determine the consequences of the lack of this protein during the interaction process with itself, host pathogens and plants. Furthermore, we constructed a *T*. *harzianum epl-1-*complemented strain (RecEpl-1-GFP) by using the Epl-1 protein fused with GFP for complementary analyses and to determine the Epl-1 cell localization and transportation dynamics under direct confrontation conditions.

Comparison of the DNA sequences flanking the promoter region of co-regulated genes might be useful to identify conserved regulatory sequences; such regions could be the binding sites for both; specific proteins and regulators^22^,^39^. Thus, we identify motifs and promoter elements that could be involved in *epl-1* regulation and/or modification in *T*. *harzianum*. Cortés *et al.*^22^ described four motifs related to MYC in the promoter of *ech42* and *prb1* genes in *T. atroviride* (MYC1, MYC2, MYC3, and MYC4). Lorito *et al.*^40^, proposed a model containing the target sequences for carbon-response regulator CreA in *Aspergillus nidulans* (which correspond to Cre1 in *T*. *harzianum*) and showed that specific MYC complex genes overlap with or are close to each other. In this study, *in silico* analyses of the promoter region of *T*. *harzianum epl-1* gene indicated one of the MYC related-motif (MYC1), which was present in four copies and always close to CreA sequence (Supplementary Figure 4); thus, it could be a target for a MYC regulation. Nonetheless, additional analyses will be necessary to confirm this. Furthermore, the presence of the stress response sequence (C4T)^22^,^28^,^29^ could explain the rapid induction of *epl-1* during the direct confrontation between *T*. *harzianum* and *S. sclerotiorum* (Supplementary Figure 4), which corresponds to a stress condition and because of the presence of inducers from the pathogen cell wall, as described for other genes^22^,^41^. Another interesting *in silico* data were the seven *O*-(beta)-*N*-acetyl-glycosylation sites in Epl-1 protein (Supplementary Figure 2) proteins from the CP family exhibit a secretion peptide signal (the first 18 peptide residues) and oligosaccharide-binding activity and residues involved in this process are well conserved^36^,^42^. This suggested that Epl-1 might play a role in polysaccharide recognition and act directly in the interaction process with different hosts. Accordingly, time lapse fluorescence microscopy indicated the predominant presence of Epl-1 in the *Trichoderma* cell wall under confrontation conditions. Furthermore, Epl-1 hyphal transportation by vesicles in a highly dynamic protein flow, being strongly induced in the presence of pathogens (Fig. 2 and Supplementary Video 1 and 2). These data are in accordance with the qPCR analyses which indicated an up-regulation of *epl-1* expression after hyphae contact in the direct confrontation assay with *S. sclerotiorum* (Supplementary Figure 10A).

Initially we did not observe phenotypic differences between wild-type and mutant *T*. *harzianumT*. *harzianum* ∆*epl-1* strains, with regard to growth (dual culture; Fig. 1) and interaction (antagonistic potential) tests (Fig. 3A1), as was reported for other *Trichoderma* species^36^,^43^. However, microscopically the differences became evident, as showed by SEM analyses. Previous studies indicated that *T*. *harzianum* has different host recognition mechanisms, and the mycoparasitism process depend on the pathogen type and its cell wall composition^34^. The *Trichoderma* mycoparasitism process involves connection to the host cell wall carbohydrates and host lectins might be involved in the recognition process^44^. Once connected, the hyphae coil tightly around the host, forming mycoparasitism-related structures (e.g., hooks and appressorium-like bodies); next, cell wall-degrading enzymes are released^45^. In this study, SEM analyses showed excessive hyphal degradation in the confrontation analyses between wild-type *T*. *harzianum* and mutant ∆*epl-1* strains, with a very well-defined degradation line in the interaction zone (but not in the wild type-wild type confrontation; Fig. 3A,B). However, hyphal degradation or debris was not observed during the confrontation between complemented strains (RecEpl-1-GFP; Fig. 3C). This finding, could be related to the non-*Trichoderma* hyphal recognition, which, as suggested by Cortes *et al.*^22^, would lead to the strong release of lytic enzymes such as (β-1,3-glucanases [*exg1*], chitinases [*nag1*, *chit33*, and *chit42*], and proteases [*papA*, *PRA1*, and *sprT*]), resulting in the digestion of the major cell wall components. This evidence can be supported by the fact that most of the analyzed mycoparasitism-related genes were up-regulated during the confrontation between wild-type and mutant ∆*epl-1 Trichoderma* strains after hyphal contact (Supplementary Figures 10 and 11). The altered expression of these genes became clear from the heat map generated from the qPCR data of *T*. *harzianum* strains that had confronted after hyphal contact (Fig. 5B), suggesting an altered perception of *Trichoderma* after coming in contact with the mutant ∆*epl-1* strain and the host pathogen.

Considering the *T*. *harzianum* Epl-1 cell localization, probable regulation, and cell wall interaction, its function in mycoparasitism process can be speculated. First, Epl-1 might act as a recognition molecule to identify its own and/or host hyphae while avoiding self-degradation. Alternatively, this protein could have a protection function, such as fish scales, from host and its own degrading enzymes and other metabolites, thereby preserving its own cell wall integrity during confrontation against prey. Gruber & Seidl-Seiboth^46^ hypothesized that self and non-self fungal cell wall degradation is regulated by substrate accessibility: cell wall protection is noted in healthy hyphae but not during mycoparasitic attack, hyphal ageing, and autolysis. They argued that the accessibility of chitin within the fungal cell wall could be the major determinant, and the accessibility of healthy hyphae might be limited by the protection conferred by cell wall proteins. According to this theory, during mycoparasitism, the prey fungus is weakened by numerous secondary metabolites as well as hydrolytic enzymes which are highly destructive for the host hyphae and leading to partial cell death, thereby increasing the accessibility of prey’s cell wall carbohydrates. This leads to the enzymatic release of oligomers, mainly oligossacharides, which in turn induce the production of even more hydrolytic enzymes and produce greater damage to the host cell wall, as was observed during the confrontation between wild-type *T*. *harzianum* and ∆*epl-1* mutant strains (Fig. 3B). Hydrophobic cell wall proteins and carbohydrate-binding proteins would be suitable candidates for affording protective functions, such as QID74 from *T*. *harzianum*^47^, which has been shown to be involved in cell protection and adherence to hydrophobic surfaces by acting like hydrophobin. Small cysteines-rich secreted proteins as well as carbohydrate-binding proteins were suggested to also be involved in aspects of the regulation of cell wall degradation and protection by binding to short oligosaccharides influencing the induction of cell wall-degrading enzymes or even by binding to chitin and masking and protecting it from degradation. Thus, Epl-1 might be a small cysteines-rich secreted protein that interacts with the cell wall and protects against enzyme degradation. Thus, the mycoparasitism-related genes could be higher induced during *Trichoderma* confrontations (Supplementary Figures 10 and 11), because the enzymes released in the interaction region that have more access to the mutant cell wall and degrade more substrate, thereby releasing more products that act as elicitors and induce the production of more enzymes that are important in mycoparasitism process^22^,^48^, such as β-1,3-exoglucanase and chitinases.

SEM analyses also showed that *T*. *harzianum* wild-type strain perfectly coiled around the *S. sclerotiorum* hyphae, as previously reported for other phytopathogens^34^,^49^, however although *T*. *harzianum* ∆*epl-1* mutant strain could recognize and parasitize the host, the coiling structures could not be detected (Fig. 4B), indicating that the lack of Epl-1 also affected the mycoparasitism interaction. This is supported by the fact that the coiling ability was recovered by the RecEpl-1-GFP strain (Fig. 4C); however, the mechanism by which Epl-1 affects the mycoparasitic hyphal coiling process is yet unknown.

Numerous signaling molecules of microbial origin that initiate plant defense responses (elicitors) have been characterized^50^. Plant cells exposed to elicitors, respond with a battery of cellular changes related to defense^51^,^52^. Vargas *et al.*^6^, showed that Sm1 and Epl-1 monomers could undergo intermolecular interactions forming dimmers, resistant to treatment with SDS/β-mercaptoethanol (and other detergents at various temperatures), suggesting a covalent interaction between these subunits (not disulfide bonds). They also suggested that the monomeric form of Sm1 and Epl-1 proteins could activate defense responses in maize leaves, and no activity was detected for the dimeric form. Thus, the plant was speculated to have specific mechanisms for distinguishing between monomeric and dimeric forms or detecting special features of the monomeric form that are not exposed when the subunits are added. Biochemical characterizations of these proteins showed that two main factors might control their oligomeric state, and thus induce their activities, the first one being glycosylation. Presence of sugar molecules would provide steric hindrance to the protein subunit association, and prevent its dimerization. Thus, the potential *O*-glycosylation (Supplementary Figure 3) observed in the *in silico* analyses in this study indicated that *T*. *harzianum* Epl-1 could have this kind of dimerization control. Another factor that could affect the oligomeric state of these proteins is the oxidation of a tryptophan residue. Tryptophan oxidation is known to be involved in the aggregation and inactivation of some proteins^51^. Vargas *et al.*^6^ suggested that the dimeric form of the Epl-1 shows a highly conserved site of tryptophan residue in its oxidized state. In this study, the analysis of this conserved site with the alignment of *T*. *harzianum* Epl-1 protein sequence showed 100% identity with *T. virens* Sm-1 (YHWSTQGQIPR) and 91% identity with *T. atroviride* Epl-1 protein. The accumulation of reactive oxygen species in plant cells was thought to stimulate the oxidation of this site and thus its aggregation, thereby altering the recognition and activation of plant responses^6^.

The activation of plant defense mechanisms occurs via a succession of events and signals that begin with the plant recognition of pathogenic/nonpathogenic microorganisms and culminates in the formation of physical barriers and the activation of chemical responses, affording protection against invasion^49^. The plant defense responses include three main mechanisms: (a) hypersensitive response (HR), (b) systemic acquired resistance (SAR) that includes induction of pathogenesis-related (PR) proteins and (c) inducted systemic resistance (ISR), which includes production of compounds such as jasmonic acid and hydrogen peroxide^53^. The mechanisms used by *Trichoderma* species to trigger plant defense responses are not completely understood; however, studies concerning differential gene expression and alterations in protein expression patterns of leaves and roots from host plants during association with *Trichoderma* species have been performed^3^,^54^,^55^. The PR protein synthesis, involving peroxidases, chitinases, β-1,3-glucanases and lipoxygenase, can be considered as one of the most obvious alterations in plant-pathogen interaction. Other parallel infection responses include increased expression of genes related to the synthesis of phytoalexins and phenylalanine ammonia lyase (PAL), along with the deposition of lignin and increased levels of salicylic acid (SA)^56^. Pereira *et al.*^57^ described the interaction between *T*. *harzianum* with host *Phaseolus vulgaris* plants in the presence or absence of the phytopathogenic fungi *F. solani* and *R. solani* and showed that the *Trichoderma* strain could promote the growth of bean plants by increasing their overall size, foliar and root area, and the number of secondary roots, as well as by modifying their root system architecture. The authors also suggested that common bean plants challenged by *T*. *harzianum* showed differential expression patterns for defense response genes, compared to those in unchallenged plants and those challenged with *F. solani* or *R. solani* alone. In this study, we analyzed the effect of Epl-1 protein in eliciting defense response genes such as *bch1*, *glu1*, *lox*, and *pod3*^57^, including a phenylalanine ammonia lyase (*pal*)-encoding gene. Our results also showed the up-regulation of *lox* and *glu1*, especially in the first 24 h after plants were challenged with *Trichoderma* strain^49^,^53^,^57^,^58^.

Interestingly, the mutant ∆*epl-1* showed almost 20-fold higher *lox* expression than that of the wild-type strain (Fig. 5). Further, a small amount of β-1,3-glucanases is synthesized and secreted into the intercellular space; with the growth of the fungal parasite or symbiont in this space, the enzyme initiates the process of fungal cell wall degradation. When the invading fungus penetrates the plant cells the vacuoles are ruptured leading to the release of enzymes that inhibit the pathogen action^59^. Interestingly, this gene was strongly induced in mutant ∆*epl-1* than in the wild-type strain (Fig. 5), indicating that Epl-1 was a cell wall protection protein. The up-regulation of *glu1* in bean plants inoculated with *T*. *harzianum* ∆*epl-1* compared to that in wild-type strain was consistent with finding in plants challenged with pathogens such as *R. solani*^49^,^53^,^57^,^58^. Thus, without the Epl-1 protein, the bean plants could recognize *T*. *harzianum* as a pathogen, since PR proteins such as β-1,3-glucanase (PR2) and chitinases (PR3), which are known to disrupt the fungal mycelial wall, can be induced by SA as well as by pathogenic attack^3^,^60^ or could also indicate a cross-talk between ISR and SAR defense pathways. The absence of Epl-1 protein in *T*. *harzianum*-plant system, found in this study, did not statistically affect the *bch1* and *pod3* gene expression, although a late *pod3* up-regulation was noted, 72 h after *T*. *harzianum* strain inoculation (data not shown). Therefore, based on *bch1* expression level, we can speculate that other CPs such as Epl-2 and Epl-3, could interact with the chitin and chitin-derived oligomers, where the chitin fragments released by the fungus, because of plant-microbe interaction would be captured by other CPs, according to their affinity^61^,^62^, thereby maintaining stable the *bch1* expression level. Salas-Marina *et al.*^63^ showed that *T. atroviride* and *T. virens* could successfully induce systemic disease resistance in tomato accompanied by increased expression levels of SA defense-related genes; however, in our study, this gene was not or slightly affected in plants inoculated with mutant ∆*epl-1* strain, suggesting that the induction of plant SA resistance system by *T*. *harzianum* strains is Epl-1 independent or that this mechanism acts on the basis of sensitivity rather than occurs because of the accumulation of PAL hormone.

Although the actual role of Epl-1 in *Trichoderma* physiology is not completely clear, the information available about this protein and the data generated in this study suggest that Epl-1 plays a fundamental role in the successful *Trichoderma* adaptation to its environment, especially during the interaction with fungal pathogens and plants. Thus, a better understanding of proteins, such as Epl-1 and their role in fungal interaction with themselves and hosts will be necessary for the improvement of strategies to combat plant pathogens and to produce crops that are more resistant to phytopathogenic organisms and to reduce the environmental pollution caused by pesticides and other chemical compounds.

## Methods

### Microorganisms and culture conditions

*T*. *harzianum* (ALL42), *F. solani* and *R. solani* isolates were provided by the Department of Biochemistry and Molecular Biology, Biochemistry Laboratory, Federal University of Goiás, Goiânia, Goiás, Brazil. *S. sclerotiorum* isolate was provided by Embrapa (Brazilian Company of Agronomic Development - Rice and Bean Unit, Santo Antônio de Goiás, Brazil), maintained with periodical sampling on potato dextrose agar (PDA), and stored at 4°C before use.

### Generation of ∆*epl-1T*. *harzianum* strains

Knockout strains were generated from *T*. *harzianum* by using primers that are listed in Table 1. For construction of the *epl-1* deletion vector, 950 bp from promoter (Epl-1 P) and 1.000 bp from terminator (Epl-1 T) flanking regions of *epl-1* were amplified using genomic DNA as template. The PCR products were digested with the appropriate restriction enzymes and cloned into a pBS (pBluescript SK+) vector (Stratagene, La Jolla, CA) containing a selection *hph* cassette (*hph* gene, conferring resistance to hygromycin, under the control of *Aspergillus nidulans gpdA* promoter and *cbh2* terminator)^71^ (Supplementary Figure 12A,B). The pBShphEpl-1 final vector (as a DNA template) and the more external primer set (Epl1*KpnI*F and Epl1*BamHI*R) were used to generate the linear *epl-1* deletion cassette. Fungal transformation was performed using protoplast generation as previously described^64^. Transformants were selected using hygromycin B (100 μg/mL) resistance. The selected transformants were analyzed using PCR and by Southern hybridization (using MSEpl-1 primers set; Table 1) to detect transformants with the pPBShphEpl-1 vector inserted into the *epl-1* gene.

### Complementation of *T*. *harzianum* mutant strain with reintegration of the *epl-1* fused with GFP-encoding genes

The Epl-1::GFP cassette was constructed according to Colot *et al.*; Goldman *et al.*; Colabardini *et al.*^65^,^66^,^67^ (Supplementary Figure 7A). The GFP signal was detected in fluorescence microscopy images (×40 magnification) under green fluorescence obtained by illuminating with 480 nm light elicited during the light flash (750 ms duration; Supplementary Figure 7B). PCR of the *epl-1* gene using the primer set RecEpl-1MS (Table 1) generated 366-bp fragments from both wild-type and complemented *T*. *harzianum* strains (no amplification from *T*. *harzianum* ∆*epl-1* mutant strain; Supplementary Figure 7C). The RT-qPCR analyses of the *epl-1* relative expression were made using the wild-type, mutant ∆*epl-1* and RecEpl-1-GFP *T*. *harzianum* strains (Supplementary Figure 7D).

### Direct confrontation assays

The antagonism of *Trichoderma* strains against the three phytopathogenic fungi (*S. sclerotiorum*, *R. solani*, and *F. solani*) was detected by taking discs of potato dextrose agar (PDA) medium from the edge of actively growing colonies of fresh fungal cultures and placing 8-cm apart on the surface of a fresh PDA plate. The plates were incubated at 25°C with a photoperiod of 12 h. The evaluation was performed when the pathogen completely covered the control plate without *Trichoderma* according to the classification proposed by Bell *et al.*^30^ with the following modification. Briefly, grade 1-*Trichoderma* completely overgrew the pathogen and covered the entire medium surface; grade 1.5-*Trichoderma* occupied 7/8^th^ of the medium surface, grade 2-*Trichoderma* occupied 6/8^th^ of the medium surface; grade 2.5-*Trichoderma* occupies 5/8^th^ of the medium surface; grade 3-*Trichoderma* occupied approximately half of the medium surface; grade 3.5-*Trichoderma* occupied 3/8^th^ of the medium surface; grade 4-*Trichoderma* occupied 1/3^th^ of the medium surface; grade 5-the pathogen completely overgrew the *Trichoderma* strain. The experiment was conducted with three repetitions for each *Trichoderma* strain. Mycelia from the interacting zones were collected by performing direct confrontation assays on PDA plates covered with sterile cellophane sheets. *T*. *harzianum* (wild-Type) and *T*. *harzianum* ∆*epl-1* were tested against *S. sclerotiorum* isolate. Agar plugs cut from growing colonies of each fungus were placed on the opposite sides in 9-cm diameter plates and incubated at 28 °C with a 12 h/12 h light/dark cycle^68^. Mycelia were collected before they contacted each other (after 3 days), when they were just touching (after 4 days), and when the interaction zone was approximately 1-cm wide (after 7 days). The confrontation assays were performed as follows: (1) *T*. *harzianum* (wt) × *T*. *harzianum* (wt) as control confrontation plate, (2) *T*. *harzianum* (wt) × *S. sclerotiorum*, (3) *T*. *harzianum* (wt) × *T*. *harzianum-*∆*epl-1*, and (4) *T*. *harzianum-*∆*epl-1 × S. sclerotiorum*.

### Dual culture tests and scanning electron microscopy analysis (SEM)

Mycelial samples and SEM analyses were performed as described in Monteiro *et al.*^69^.

### Fluorescence microscopy

Fresh spores from *T*. *harzianum* RecEpl-1-GFP strain culture and a disc from PDA medium (5-mm diameter) containing *S. sclerotiorum* mycelium were inoculated in PDA medium for hyphal development. Small pieces of PDA medium containing *T*. *harzianum* and *S. sclerotiorum* mycelium were placed on the opposite sides of the microscope plate covered with a very thin water/agar (2% w/v) film and placed in a chamber for overnight growth at 25°C and 60% humidity. A microscopy plate without *S. sclerotiorum* was used as a control. The *Trichoderma* development and the Epl-1-GFP dynamics were monitored in the presence or absence of *S. sclerotiorum* by using the BioStation IMq (Nikon, Japan) cell incubator and BioStation software version 2.1. The samples were monitored during 24 h at 32 °C and 60% humidity. The images were captured every 10 min at ×20 and ×40 magnification, in the light field and green fluorescence, which was obtained by illuminating with 480 nm light, elicited during the light flash (duration 750 ms). The images were edited, and the movies were obtained using ImageJ Software version 1.48 (National Institute of Health, United States).

### DNA/RNA manipulations

All DNA manipulations were performed by standard techniques^74^. The Southern hybridization was performed as described in Cardoza *et al.*^10^. The probe was produced from the PCR-amplified fragment by using MSEpl1 primers set (Table 1). Total fungal RNA was extracted from the mycelia of each sample by using TRIzol® RNA kit (Invitrogen Life Technologies, Carlsbad, CA, USA), according to the manufacturer’s instructions. RNA concentrations were determined by spectrophotometry at OD 260/280, and RNA integrity was verified using 1% agarose gel electrophoresis. Manipulations and analyses of RNA from bean plants was performed as described in Pereira *et al.*^70^

### qRT-PCR analysis

#### Expression analysis of *T*. *harzianum* mycoparasitism-related genes

Approximately, 0.5 μg of RNA from the direct confrontation assay (described in section 2.3) was isolated and treated with DNase I (Thermo Scientific, Fermentas, Vilnius, Lithuania) and reverse-transcribed to cDNA by using the First strand cDNA kit Maxima™ Synthesis according to manufacturer’s instructions. cDNAs were diluted 1:50 and used for real-time PCR analysis in the Bio-Rad CFX96™ System by using SsoFast™ Eva Green® Supermix (Bio-Rad, San Francisco, CA, USA) for signal detection in accordance with the manufacturer’s instructions. The actin gene (*act*) was used as an endogenous control (housekeeping)^71^. Twelve genes were analyzed: *epl-1*; 1,3-β-exoglucanase (*exg1*); manosidase (*gh92*); α-1,3-glucosidase (*mutAW*); a*cid phosphatase*; phytase (*phyAT*); chitinase (*nag1*); chitinase 42 (*chit42*); chitinase 33 (*chit33*); aspartyl protease (*papA*); trypsin-like protease (*PRA1*); and serine protease (*sprT*). Primers sequences are described by Steindorff *et al.*^72^. The following amplification program was used: 95 °C for 10 min followed by 39 cycles of 95 °C for 10 s, and 60 °C for 30 s followed by a dissociation curve of 60 °C to 95 °C with an increment of 0.5 °C for 10 s. Gene expression values were calculated according to the 2^−ΔΔCt^ method^73^ by using *T*. *harzianum* x *T. harzianum* (no contact) as a reference sample. Gene expression analyses were performed using three technical and biological replicates. Data analysis was performed using GraphPad Prism v 5.1 software. The results were compared by one-way analysis of variance (ANOVA) with Bonferroni test (α = 5%) to analyze the differences between the related conditions and the reference sample.

#### Expression analysis of defense genes in bean plants

*P. vulgaris* growth was assessed in triplicate using 10 plants for each experimental replicate under two conditions (with *T*. *harzianum* WT-control condition; with *T*. *harzianum* ∆*epl-1*), totaling 60 plants. Seeds of common bean (*P. vulgaris*), were surface sterilized by immersion in sodium hypochlorite solution at 1% of active chlorine for 7 min, followed by three autoclaved water washes, 1 min each. After sterilization, the seeds were germinated in sterile glass Petri dishes containing 2% agar and 2% glucose, which were previously autoclaved. The seeds were kept in a germination chamber BOD at 28°C with a photoperiod of 16 h light/8 h dark. After four days, the bean seeds were placed in a hydroponic cultivation system^3^. Adapted 50 mL conical tubes were placed in 250 mL flasks containing 50 mL of Murashige and Skoog (MS), and Gamborg’s Vitamin Solution prepared according to the manufacturer’s instruction (Sigma-Aldrich, Co., Wisconsin, USA), and sealed with plastic wrap. This system was developed to allow germination, growth, and interaction of bean plants with *Trichoderma* strains, and the seedling contacted with the medium with partial immersion. Seeds were again grown with a photoperiod of 16 h light for 4 days in a germination chamber BOD with gentle agitation. Subsequently, the medium was replaced by fresh MS medium plus 0.05% sucrose and inoculated with 10^7^ spores/mL of each *Trichoderma* strain directly on the seedlings roots. The leaves and roots were collected 24, 48, and 72 h after inoculation, and stored at −80 °C until RNA extraction. A group of plants grown in the absence of fungal isolates was used as a control.

## Additional Information

**How to cite this article**: Vieira Gomes, E. *et al.* The Cerato-Platanin protein Epl-1 from *Trichoderma harzianum* is involved in mycoparasitism, plant resistance induction and self cell wall protection. *Sci. Rep.* **5**, 17998; doi: 10.1038/srep17998 (2015).

## Supplementary Material

Supplementary Information

Supplementary Video 1

Supplementary Video 2

## Figures and Tables

**Table 1:** Primers for *T. harzianum* Transformation and Transformants Screening.

PRIMER	TARGET REGION	SEQUENCE	AMPLICON (bp)
Epl1KpnIF	Epl-1 Promoter	ATGGGTACCGCTGTGGCGATGCTTCTCCAAGT	
Epl1XhoIR	Epl-1 Promoter	ATGCTCGAGTTTGCCTCCTCCCTTC	950
Epl1HindIIIF	Epl-1 Terminator	ATGAAGCTTGGTTATTGCATTGCATTGTG	
Epl1bBamHIR	Epl-1 Terminator	ATGGGATCCTCCTTTGCCATTGCAGAATC	1000
MSEpl1F	Epl-1 ORF	TTGGCGATGGCTGGATACTACGAT	WT: 1090
MSEpl1R	Epl-1 ORF	TGCCACGTCGATCAGCTTACAAGA	∆*epl-1*: 3457
HphF	Hygromycin ORF	GCGGAGGCCATGGATGCGAT	
HphR	Hygromycin ORF	GCGCTTCTGCGGGCGATTTG	596
RecEpl-1MSF	Epl-1 ORF	ATGCAATTGTCCAGCCTC	
RecEpl-1MSF	Epl-1 ORF	CTTGACAGCGACCTGGC	466
