# Supplementary material for: The Cerato-Platanin protein Epl-1 from Trichoderma harzianum is involved in mycoparasitism, plant resistance induction and self cell wall protection
Source: Sci Rep. 2015 Dec 9;5:17998. doi: 10.1038/srep17998 (PMC4673615; doi:10.1038/srep17998)
Supplement: Supplementary Information [file srep17998-s1.pdf]

Supplementary Material

The Cerato-Platanin protein Epl-1 from *Trichoderma harzianum* is involved in mycoparasitism, plant resistance induction and self cell wall protection

Eriston Vieira Gomes[1], Mariana do Nascimento Costa[1], Renato Graciano de Paula[1], Rafael Ricci de Azevedo[2], Francilene Lopes da Silva[3], Eliane F. Noronha[3], Cirano José Ulhoa[4], Valdirene Neves Monteiro[5], Rosa Elena Cardoza[6], Santiago Gutiérrez[6], Roberto Nascimento Silva[1*]

1 - Department of Biochemistry and Immunology, Ribeirão Preto Medical School, University of São Paulo, Ribeirão Preto, SP, Brazil.

2 - Department of Molecular and Cellular Biology and Pathogenic Bioagents, Ribeirão Preto Medical School, University of São Paulo, Ribeirão Preto, SP, Brazil.

3 - Department of Cellular Biology, University of Brasilia, Brasília, Distrito Federal, Brazil.

4 - Department of Biochemistry and Cellular Biology, Biological Sciences Institute, Federal University of Goias, Goiânia, Goiás, Brazil.

5 - Department of Biochemistry, State University of Goias, Anápolis, Goiás, Brazil.

6 - Department of Microbiology, University School of Agricultural Engineers, University of León, Ponferrada, Spain.

***Correspondence:**

Dr. Roberto do Nascimento Silva

University of São Paulo

Ribeirão Preto Medical School

Department of Biochemistry and Immunology

3900, Bandeirantes Av.

14049-900

Ribeirão Preto, SP

rsilva@fmrp.usp.br

1 - Supplementary Figures:

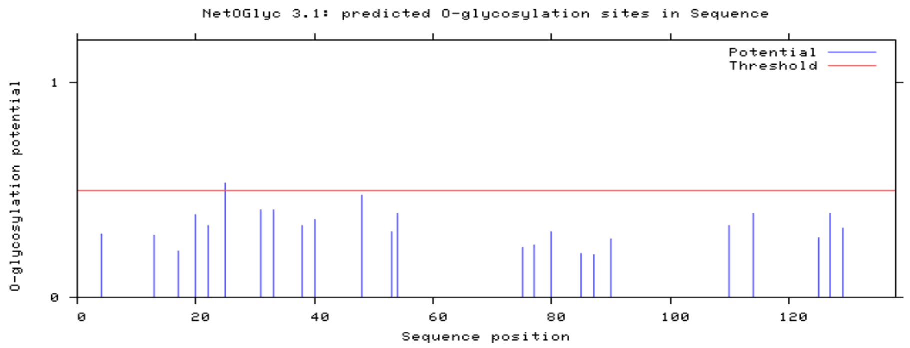

Supplementary Figure 1: Analysis of potential *O*-glycosylation sites in *T. harzianum* Epl-1 protein sequence. The red horizontal line indicates the threshold potential *O*-glycosylation. The blue vertical lines indicate the position of the site in the protein sequence. Sites with blue vertical lines which cross the red threshold line have potential *O*-glycosylation.

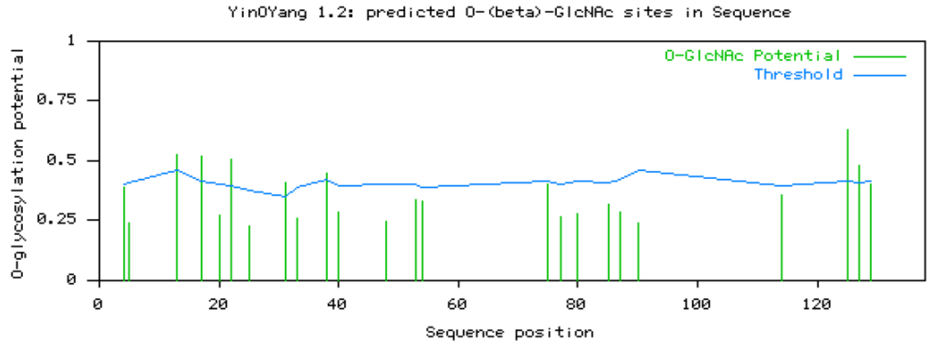

Supplementary Figure 2: Analysis of potential *O*-β-*N*-Acetil-Glicosilation sites in *T. harzianum* Epl-1 protein sequence. The horizontal blue line indicates the threshold potential *O*-β-*N*-Acetyl glycosylation. The green vertical lines indicate the position of the site in the protein sequence. Sites with green vertical lines exceeding the threshold blue line have a potential of *O*-β-*N*-Acetyl glycosylation.

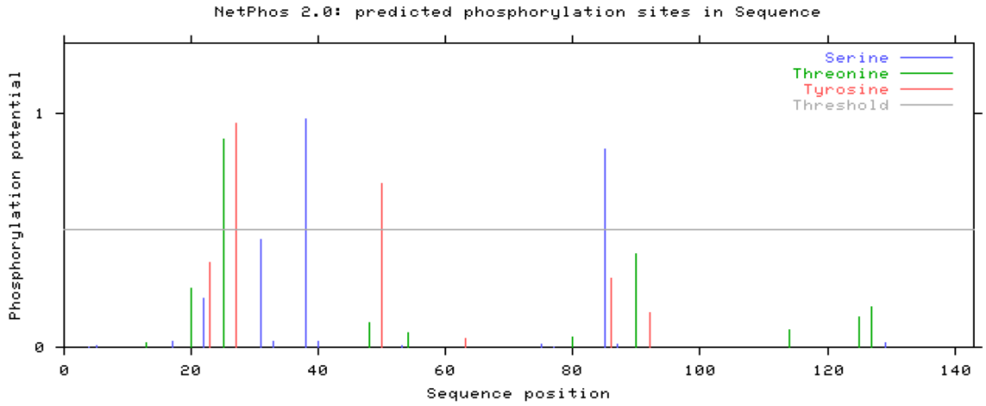

Supplementary Figure 3: Analysis of potential phosphorylation sites on *T. harzianum* Epl-1 protein sequence. The horizontal gray line indicates the threshold phosphorylation potential. The vertical colored lines indicate the potential amino acid; the position of the potential amino acid in the protein sequence. Sites with vertical lines cross the threshold line present potential phosphorylation.

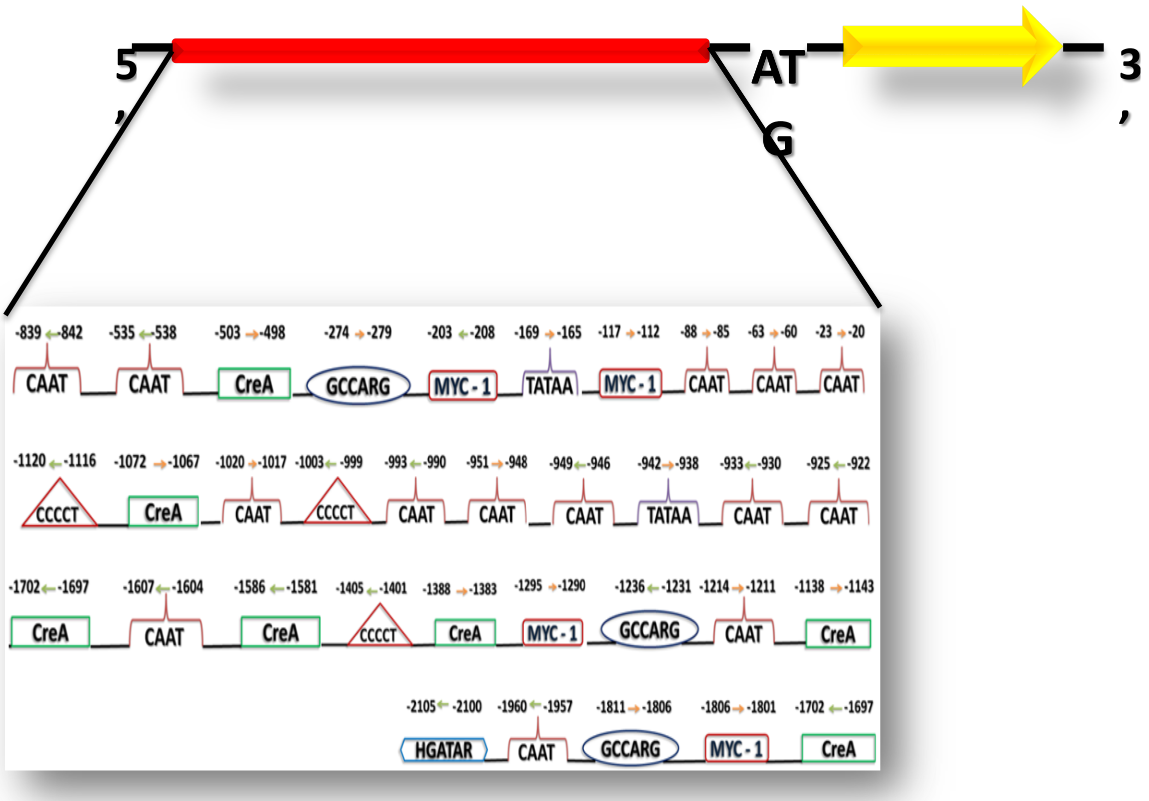

Supplementary Figure 4: Representation of predicted regulatory motifs in the promoter region of *T. harzianum epl-1* gene. The numbers indicate the position relative to the ATG translation start codon. Arrows indicate the orientation of the motif in the sense (5 '⟶ 3') and antisense strands (5 '⟵ 3') respectively. **CAAT box** and **TATAA box** – transcription initiation sites; **MYC-1** – Mycoparasitism Response Element -1; **CreA** – Carbon response regulator; **GCCARG** – pH regulatory protein site; **CCCCT** – Stress response elements; **HGATAR** – Global nitrogen regulation.

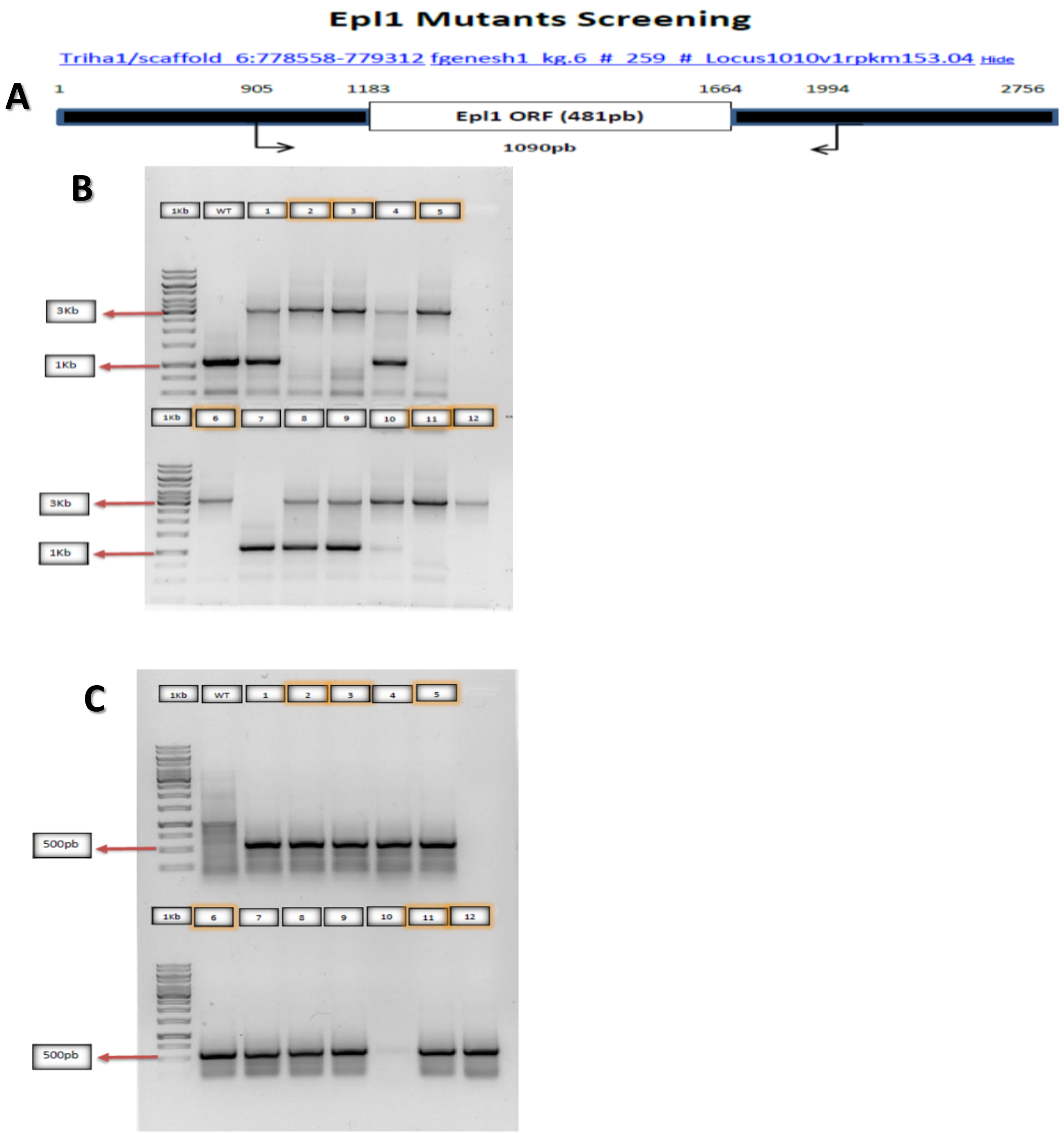

Supplementary Figure 5: Transformants screening scheme. **A** – Schematic representation of the genomic region containing the *epl-1* gene and the respective annealing sites of mutant screening primers set (MSEpl-1) (arrows). **B** – Mutant screening: agarose gel electrophoresis of *epl-1* gene PCR amplification. WT - *T. harzianum* wild type (1090 bp); 1 – 12- Screening of mitotically stable Δ*epl-1* transformants (3457bp). **C** - Mutant screening agarose gel electrophoresis of *hph* gene PCR amplification. WT - *T. harzianum* wild type (no amplification); 1 – 12- Screening

of mitotically stable Δ*epl-1* transformants (600bp). Individuals marked with orange rectangle were selected for further analysis; (1kb) Molecular weight marker.

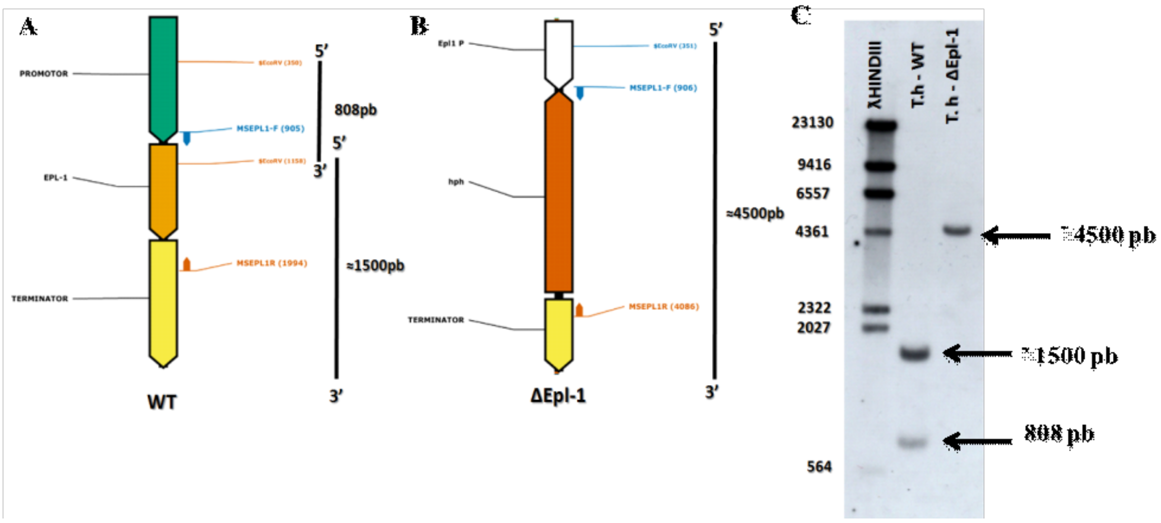

Supplementary Figure 6: Southern Blot analysis. **(A)** Representation of *epl-1* encoding gene in *T. harzianum* Wild Type strain with *EcoRV* restriction sites, the fragments formed after digestion with its respective size (vertical lines in black) and MSEpl-1 primers annealing sites for probe construction and hybridization; **(B)** Representation of *epl-1* deletion cassette with *EcoRV* restriction sites. The fragments formed after digestion with their respective sizes (vertical black lines) and MSEpl-1 primers annealing sites for probe hybridization; **(C)** Southern Blot Analysis of total DNA from *T. harzianum* Wild Type (*T.h* – WT) and *T. harzianum* Epl-1 deleted mutant (*T. h* - Δ*Epl-1*) digested with *EcoRV* endonuclease; (λ*HindIII*) Size Markers.

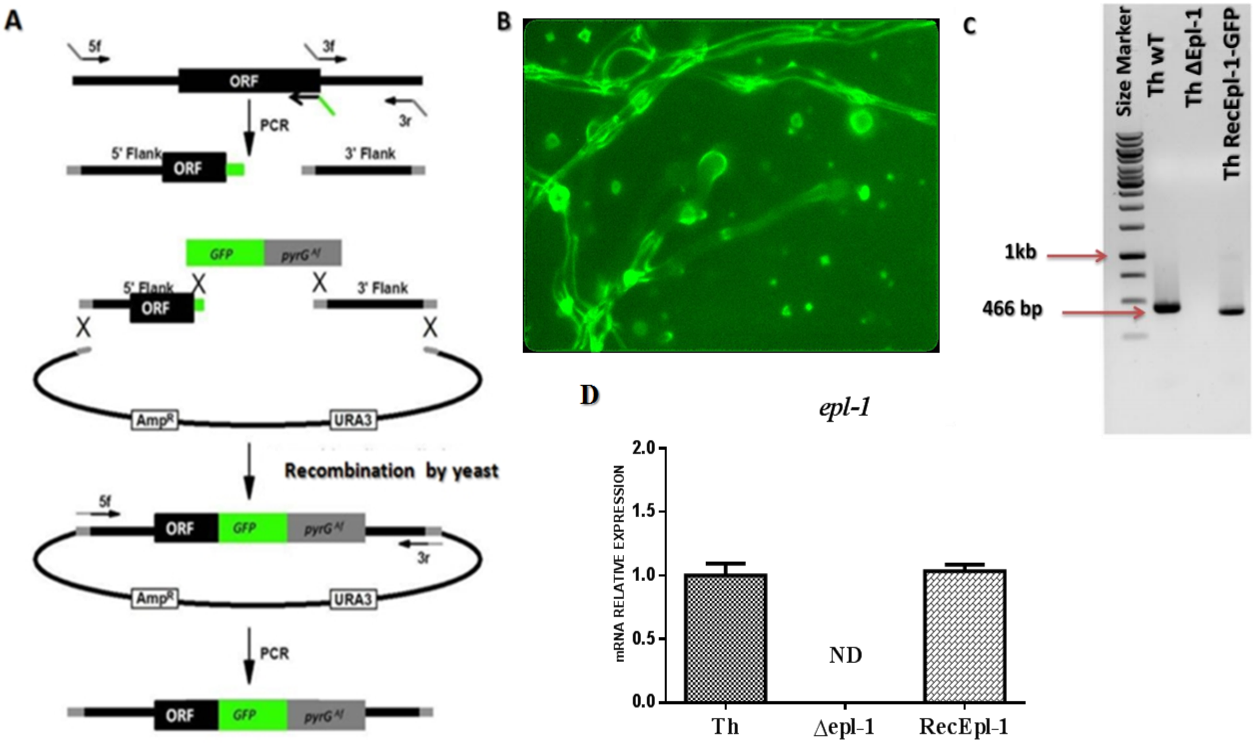

Supplementary Figure 7: *T. harzianum* RecEpl-1-GFP strain construction. **A** - Schematic representation of the Epl-1 recover cassette fused with GFP construction. **B -** *T. harzianum* RecEpl-1-GFP strain fluorescence microscopy, 40x magnification. **C** – agarose gel electrophoresis of *epl-1* gene PCR amplification. WT - *T. harzianum* wild type strain (466 bp); ThΔEpl-1 – *T. harzianum* Δ*epl-1* strain (no amplification product); Th RecEpl-1-GFP – *T. harzianum epl-1* complemented strain (466 bp). Size Marker – 1kb Molecular weight marker. **D -** Relative Expression levels (linear) of *epl-1* gene in different *T. harzianum* strains. (Th) *T. harzianum* (WT); (Δepl1) *T. harzianum* Δ*epl1*; (RecEpl-1) *T. harzianum* RecEpl-1-GFP; (ND) No detected.

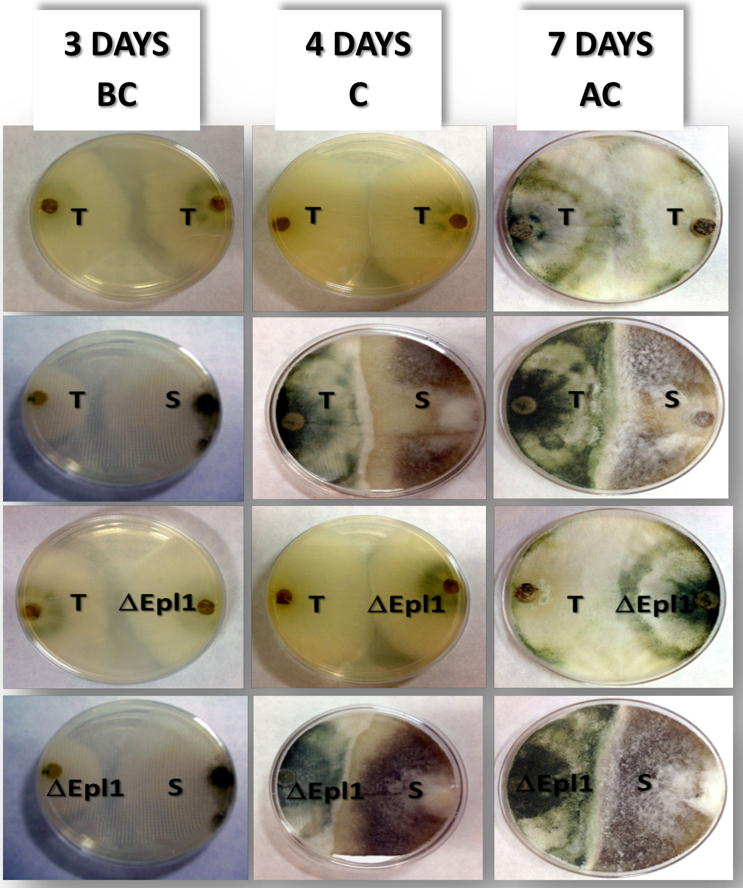

Supplementary Figure 8: Direct Confrontation Assay. **(T)** *T. harzianum* wild type; (ΔEpl-1) *T. harzianum Δepl-1*; (S) *S. sclerotiorum*; BC – Before hyphae contact; C – hyphae contact; AC – After hyphae contact.

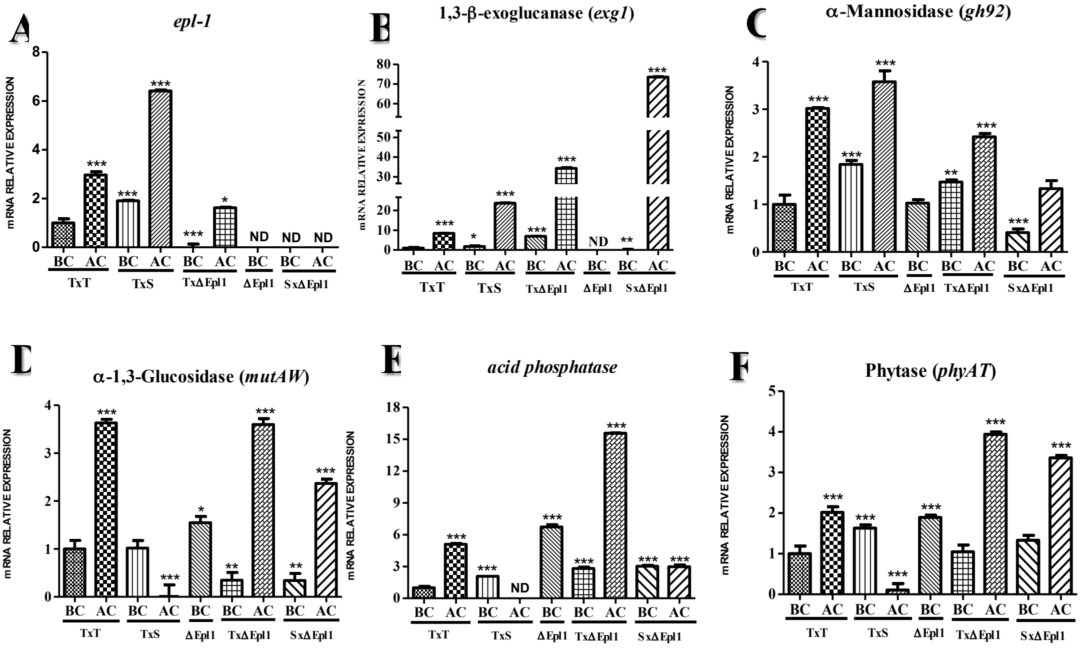

Supplementary Figure 9: Relative Expression levels (linear) of *T. harzianum* mycoparasitism-related genes in direct confrontation assay. **(T)** *T. harzianum* (WT); (S) *S. sclerotiorum*; (ΔEpl1) *T. harzianum* Δ*epl1*; (BC) before hyphae contact; (AC) after hyphae contact; (ND) No detected. The data were presented using the $2^{-\Delta\Delta Ct}$ method. * $p<0.05$, ** $p<0.01$, *** $p<0.001$.

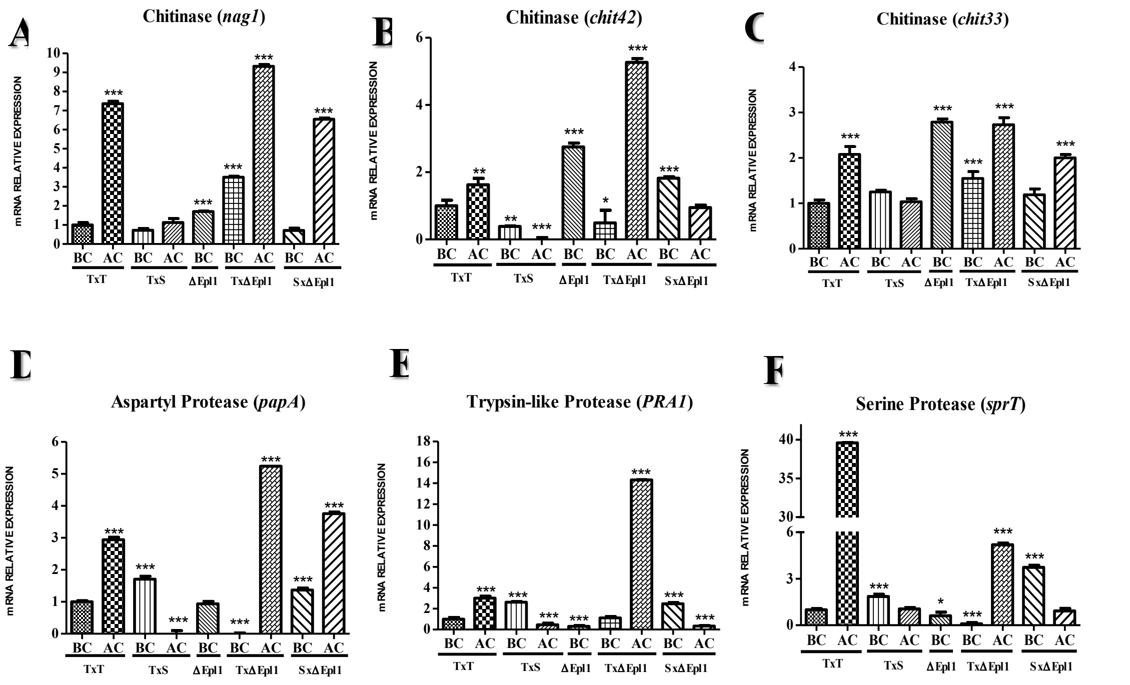

Supplementary Figure 10: Relative Expression of *T. harzianum* mycoparasitism-related genes in direct confrontation assay. **(T)** *T. harzianum* (WT); **(S)** *S. sclerotiorum;* **(ΔEpl1)** *T. harzianum* Δ*epl1*; **(NC)** no hyphae contact; **(C)** with hyphae contact; **(ND)** No detected. The data were presented using the $2^{-\Delta\Delta Ct}$ method.* p<0.05, ** p<0.01, *** p<0.001.

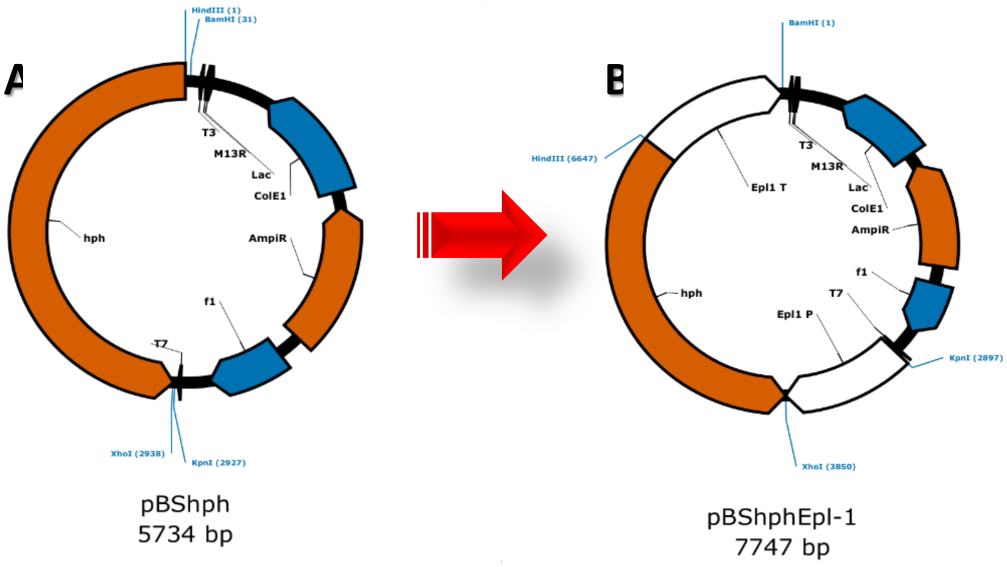

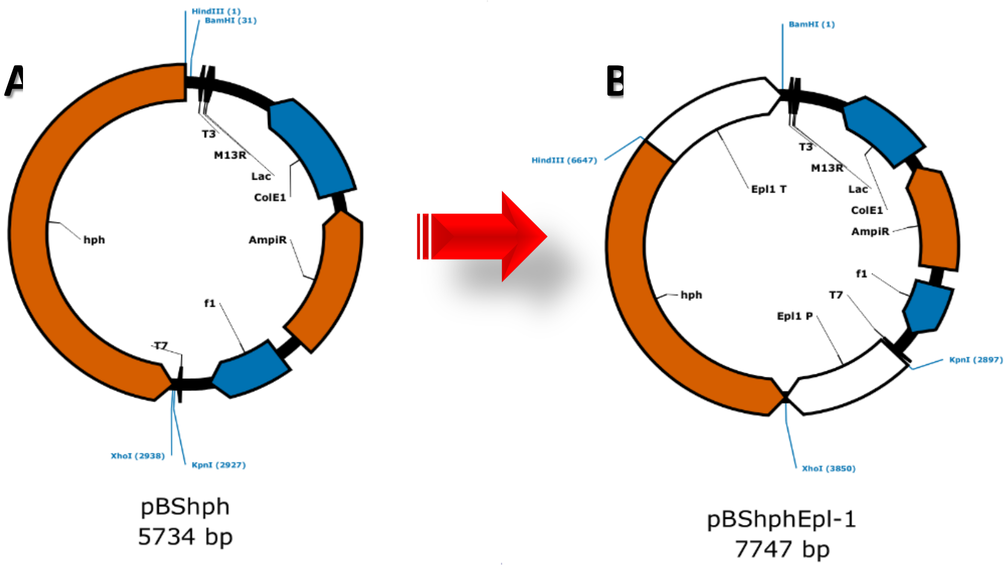

Supplementary Figure 11: Construction of Epl-1 deletion vector. **A** - pBluescript SK+ vector with selection *hph*-cassette (pBShph) with its respective restriction sites. B – Complete pBShphEpl-1 deletion vector with promoter and terminator *epl-1* region in its respective cloning sites.

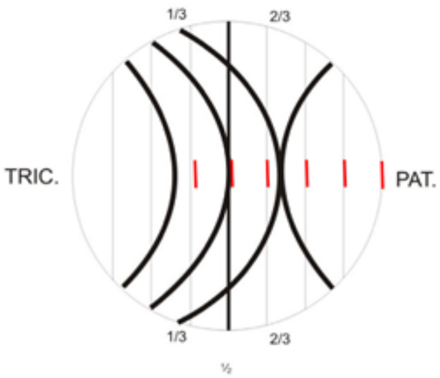

Scores	Classification
Scores < 3	Efficient
Scores = 3	Moderate
Scores > 3	Inefficient

Supplementary Figure 12: Schematic Bell et al., 1982 modified method, to classify *Trichoderma* strains in antagonistic activity assay in plate. TRIC. – *T. harzianum* strains. PAT. – Pathogen strains.

2 - Supplementary Videos Legends:

Supplementary Video 1: Fluorescence microscopy of *Trichoderma harzianum* RecEpl-1-GFP strain hyphae in 20x optical magnification.

Supplementary Video 2: Fluorescence microscopy of *Trichoderma harzianum* RecEpl-1-GFP strain hyphae in 40x optical magnification.
